# Topologically protected surface states in a centrosymmetric superconductor β-PdBi_2_

**DOI:** 10.1038/ncomms9595

**Published:** 2015-10-13

**Authors:** M. Sakano, K. Okawa, M. Kanou, H. Sanjo, T. Okuda, T. Sasagawa, K Ishizaka

**Affiliations:** 1 Department of Applied Physics and Quantum-Phase Electronics Center (QPEC), The University of Tokyo, Tokyo 113-8656, Japan; 2 Materials and Structures Laboratory, Tokyo Institute of Technology, Kanagawa 226-8503, Japan; 3 Hiroshima Synchrotron Radiation Center, Hiroshima University, Higashi-Hiroshima 739-0046, Japan

## Abstract

The topological aspects of electrons in solids can emerge in real materials, as represented by topological insulators. In theory, they show a variety of new magneto-electric phenomena, and especially the ones hosting superconductivity are strongly desired as candidates for topological superconductors. While efforts have been made to develop possible topological superconductors by introducing carriers into topological insulators, those exhibiting indisputable superconductivity free from inhomogeneity are very few. Here we report on the observation of topologically protected surface states in a centrosymmetric layered superconductor, β-PdBi_2_, by utilizing spin- and angle-resolved photoemission spectroscopy. Besides the bulk bands, several surface bands are clearly observed with symmetrically allowed in-plane spin polarizations, some of which crossing the Fermi level. These surface states are precisely evaluated to be topological, based on the *Z*_2_ invariant analysis in analogy to three-dimensional strong topological insulators. β-PdBi_2_ may offer a solid stage to investigate the topological aspect in the superconducting condensate.

## Abstract (web summary)

Materials possessing topologically non-trivial electronic surface states are predicted to host exotic Majorana fermion excitations in the superconducting state. Here, the authors demonstrate the existence of topologically-protected surface states in the centrosymmetric layered superconductor β-PdBi2.

## Graphical abstract

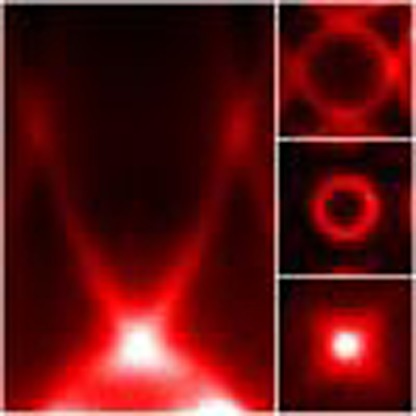

Topological insulators are characterized by the non-trivial *Z*_2_ topological invariant acquired when the conduction and valence bands are inverted by spin–orbit interaction (SOI), and the gapless surface state appears^1^,^2^,^3^. This topologically non-trivial surface state possesses the helical spin polarization locked to momentum, and is expected to host various kinds of new magneto-electric phenomena. Especially, the ones realized with superconductivity are theoretically investigated as the candidates for topological superconductor^2^,^3^,^4^, whose excitation is described as Majorana Fermions, that is, the hypothetical particles originating from the field of particle physics^5^,^6^,^7^,^8^. Experimentally, several superconductors developed by utilizing topological insulators are reported thus far, such as Cu-intercalated Bi_2_Se_3_ (refs 9, 10, 11, 12), In-doped SnTe^13^ and *M*_2_Te_3_ (*M*=Bi, Sb) under pressure^14^,^15^. While the previous studies of point-contact spectroscopy on Cu_*x*_Bi_2_Se_3_ (refs 9, 10) and In-SnTe^13^ suggest the existence of Andreev bound states thus raising the possibility of topological superconductivity, the scanning tunnelling microscope/spectroscopy reports the simple s-wave-like full superconducting gap^16^. Theoretically, this contradiction has been discussed in terms of the possible peculiar bulk odd-parity pairing^17^, which awaits experimental verifications by various probes^18^,^19^. However, partly due to the inhomogeneity effect accompanied by doping or pressurizing, the unambiguous clarification of superconducting states in doped topological insulators has been hindered until now. The half-Heusler superconductor RPtBi (R: rare earth) is another class of material recently reported as a candidate for topological superconductors^20^,^21^. Practically, however, its low critical temperature (*T*_c_) of *T*_c_<2 K and the noncentrosymmetric crystal structure without a unique cleavage plane may pose some difficulties for its further investigation.

In this work, we introduce a superconductor β-PdBi_2_ with a centrosymmetric tetragonal crystal structure of space group I4/mmm^22^,^23^,^24^ as shown in Fig. 1a. It has a much simpler structure compared with the related noncentrosymmetric superconductor α-PdBi, recently being discussed as a possible topological superconductor^25^,^26^. Pd atoms, each of them located at the centre of the square prism of eight Bi atoms, form the layered body-centred unit cell. PdBi_2_ layers are stacked in van der Waals nature, making it a feasible compound for cleaving. We investigate the electronic structure of β-PdBi_2_ using (spin-) angular-resolved photoemission spectroscopy, (S)ARPES. With the large single crystals of good quality, exhibiting the high residual resistivity ratio (∼14) and a clear superconducting transition at *T*_c_=5.3 K, several spin-polarized surface states are clearly observed in addition to the bulk bands. On the basis of the relativistic first-principles calculation on bulk and the slab calculation on surface, we find that the observed surface states can be unambiguously interpreted to be topologically non-trivial.

## Results

### Bulk and surface band structures

**Figure 1:**
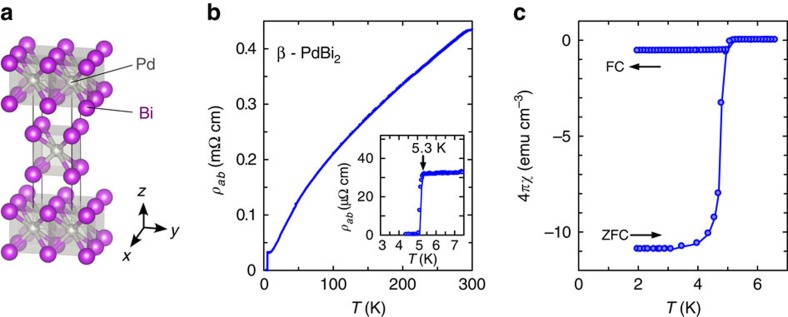
Basic properties of superconductor β-PdBi_2_. (**a**) Crystal structure of superconductor β-PdBi_2_. *x*, *y* and *z* axes are taken along the body-centred tetragonal crystal orientation. (**b**) In-plane electrical resistivity (*ρ*_*ab*_) as a function of temperature (*T*). The inset shows *ρ*_*ab*_ near the critical temperature (5.3 K). (**c**) Magnetic susceptibility (*χ*) as a function of *T*, recorded under the field-cool (FC) and zero-field-cool (ZFC) conditions. The magnetic field of 10 Oe was applied along the direction of the *c* axis.

**Figure 2:**
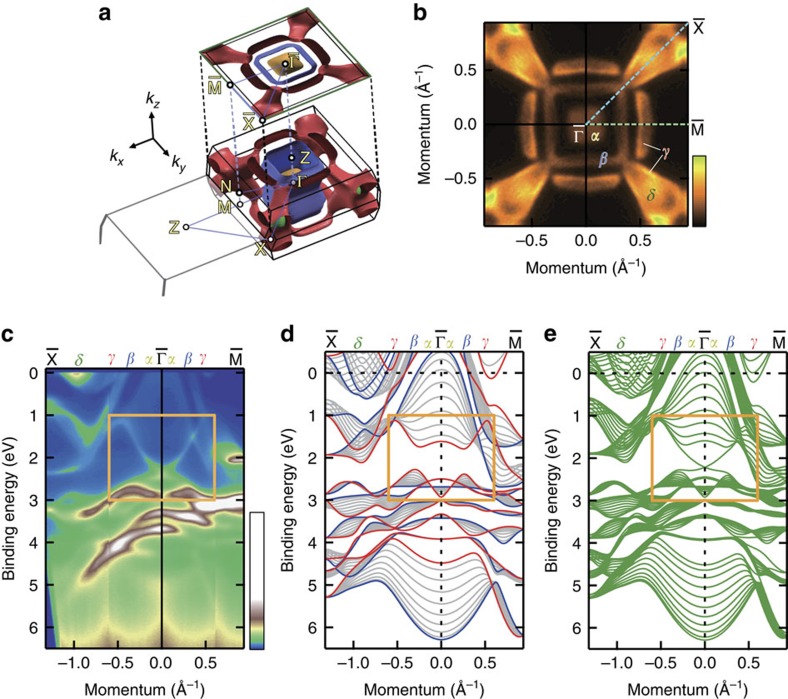
Electronic structure of β-PdBi_2_. (**a**) Calculated Fermi surfaces shown with the first Brillouin zone. *k*_*x*_, *k*_*y*_ and *k*_*z*_ axes for the crystal momentum space are depicted. Γ, Z, N, X and M are the high-symmetry points. The square plane represents the two-dimensional (2D) projected surface Brillouin zone with 2D high-symmetry points, 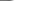, 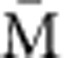 and 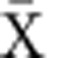. (**b**) Four-fold symmetrized Fermi surface recorded by angular-resolved photoemission spectroscopy (ARPES). The image is obtained by integrating intensities in the energy window of ±8 meV at the Fermi level. The colour scale indicates the intensity. Two electron-like and two hole-like Fermi surfaces are denoted by *α*, *β* and *γ*, *δ*, respectively. (**c**) ARPES image recorded along 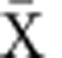
–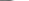 and 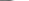
–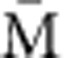 cuts, shown as the light-blue and -green broken lines in **b**, respectively. The colour scale indicates the intensity. (**d**) Calculated bulk band dispersions projected onto 2D surface Brillouin zone. Blue (red) curves correspond to *k*_*z*_=0 (2*π*/*c*). (**e**) Surface band dispersions obtained by slab calculation of 11 PdBi_2_ layers. Orange rectangles in **c**–**e** indicate the region where the surface Dirac cone appears.

Here we present the ARPES result obtained using the single-crystalline β-PdBi_2_. The resistivity and magnetic susceptibility of the sample as shown in Fig. 1b,c clearly indicate the sharp superconducting transitions. The band structure of β-PdBi_2_ observed by ARPES is shown in Fig. 2b,c. For simply describing the (S)ARPES results hereafter, we use the projected two-dimensional (2D) surface Brillouin zone depicted in Fig. 2a by a green square. The projected high-symmetry points are 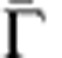, 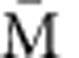 and 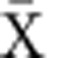, and we define *k*_*x*_ as the momentum along 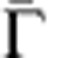
–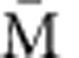. The ARPES image in Fig. 2c is recorded along 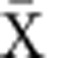
–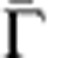 and 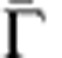
–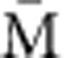, respectively. Bands crossing the Fermi level (*E*_F_) are predominantly derived from Bi 6*p* components with large dispersions from the binding energy (*E*_B_) of *E*_B_∼6 eV to above *E*_F_. On the other hand, bands mainly consisting of Pd 4*d* orbitals are located around *E*_B_=2.5∼5 eV with rather small dispersions. Near *E*_F_, two hole bands (*α*, *β*) and one electron band (*γ*) are observed along 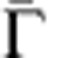
–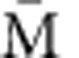, whereas for 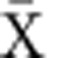
–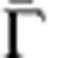, the large ARPES intensity from another electron band (*δ*) is additionally observed. As we can see in Fig. 2b, the experimental Fermi surface mapping mostly well agrees with the 2D projection of the calculated bulk Fermi surfaces (Fig. 2a).

To compare with ARPES, the calculation of bulk band dispersions projected into 2D Brillouin zone is shown in Fig. 2d. Considering that the ARPES intensity includes the integration of finite *k*_*z*_-dispersions due to the surface sensitivity, the overall electronic structure is in a good agreement with the calculation; nevertheless, several differences can be noticed. The most prominent one appears in the orange rectangles in Fig. 2c,d. A sharp Dirac-cone-like dispersion is experimentally observed where the calculated bulk bands show a gap of ∼0.55 eV around the 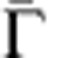 point. To confirm its origin, we performed a slab calculation for 11 PdBi_2_ layers (Fig. 2e). Apparently, a Dirac-cone-type dispersion appears in the gapped bulk states, showing a striking similarity to ARPES (Fig. 2c). It clearly presents the surface origin of this Dirac-cone band.

**Figure 3:**
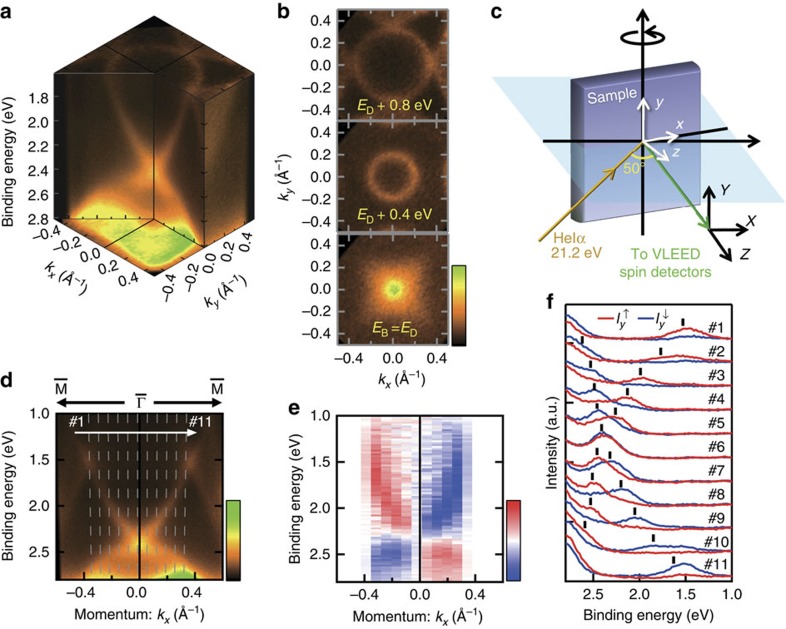
Band dispersion and spin polarization of surface Dirac-cone band. (**a**) Close-up of the observed surface Dirac-cone dispersions. (**b**) Constant-energy cuts at the binding energies (*E*_B_) of *E*_B_=*E*_D_+0.8 eV, *E*_D_+0.4 eV and *E*_D_ (=2.41 eV), respectively, where *E*_D_ is the band crossing point of the surface Dirac cone. The colour scale indicates the intensity. (**c**) Schematic of spin- and angular-resolved photoemission spectroscopy (SARPES) experimental geometry using the HeIα light source (21.2 eV) and very-low-energy electron diffraction (VLEED) spin detectors. (**d**) Intensity image of the surface Dirac cone along 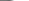
–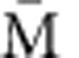. The colour scale indicates the intensity. Grey lines (#1–11) represent the measurement cuts for energy distribution curves (EDC) shown in **f**. (**e**) Spin-resolved image of the surface Dirac-cone dispersions for spin *y*-component. The colour scale indicates the spin polarization *P*_*y*_, from *P*_*y*_=−1 (blue) to *P*_*y*_=+1 (red). (**f**) Spin-resolved EDCs for momenta #1–11 as shown in **d**, respectively. Red (blue) curves show the spin-up (spin-down) component of the intensity, 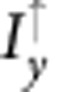 (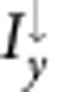). The black markers denote the peak positions of the EDC in **d**.

Now we focus on the observed surface Dirac-cone band. The close-up of the surface Dirac cone is demonstrated in Fig. 3a, indicating its crossing point at *E*_B_=*E*_D_=2.41 eV (*E*_D_: the energy of Dirac point where the bands cross each other). Such a clear Dirac-cone-shaped band strongly reminds us of the helical edge states in three-dimensional (3D) strong topological insulators. We can see the very isotropic character of surface Dirac cone in its constant-energy cuts (Fig. 3b), appearing as the perfectly circular-shaped contour even at *E*_B_=*E*_D_–0.8 eV with a large momentum radius of 0.3 Å^−1^. It is in contrast to the warping effect often appearing in trigonal strong topological insualtors^27^,^28^. The spin polarization of surface Dirac cone is also directly confirmed by SARPES experiments as depicted in Fig. 3c (ref. 29). Figure 3e,f shows the results for the *y*-component spin, measured along *k*_*x*_ (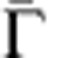
–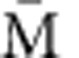). Because of C_4v_ symmetry, *x*- and *z*-components are forbidden (Supplementary Note 1; Supplementary Fig. 1). The red (blue) curves in Fig. 3f, indicating the energy distribution curves of spin-up (-down) components, clearly show the spin-polarized band dispersions. As easily seen in the SARPES image (Fig. 3e), the spin polarization with spin-up (spin-down) for negative (positive) dispersion of surface Dirac cone is confirmed. The observed spin-polarized surface Dirac cone thus presents a strong resemblance to the helical surface state in strong topological insulators.

### Analysis of the topological invariant

**Figure 4:**
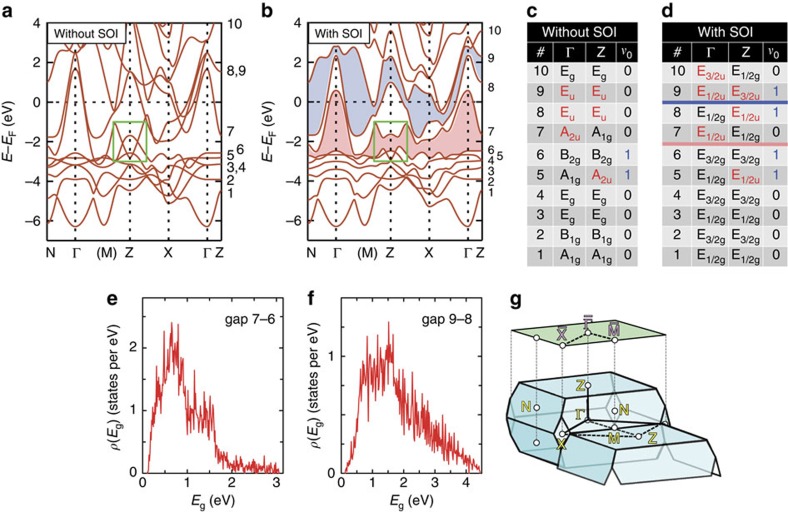
Analysis of parity and topological invariant for valence bands. (**a**,**b**) First-principles band calculations without and with spin–orbit interaction (SOI), respectively. Valence bands are numbered by the energy (*E*) at the Z point, as shown in the right side of the panels. The green rectangles indicate the energy region where the surface Dirac cone appears. Pink (blue)-shaded area in **b** shows gap 7−6 (gap 9−8) induced by SOI. (**c**,**d**) Lists of the topological invariant *ν*_0_ and the symmetries of wavefunctions at the Γ and Z points without and with SOI, respectively. The left-end columns (#) indicate the number of the valence bands as given in **a** and **b**, respectively. The symmetries indicated with red (black) have the odd (even-) parity. *ν*_0_=1 indicates the topologically non-trivial band-inverted state. The pink (blue) line in **d** denotes the gap 7−6 (gap 9−8). (**e**,**f**) The distribution of the direct gap (*E*_g_) for gap 7−6 and gap 9−8, respectively, obtained by the band calculation. (**g**) The blue solid (green plane) indicates the three (two)-dimensional Brillouin zone with high-symmetry points Γ, Z, N, X and M (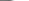, 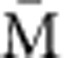 and 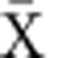). Γ, Z, N and X are the three-dimensional time-reversal invariant momenta (TRIM).

To evaluate whether the observed surface state is topologically non-trivial, we derive the *Z*_2_ invariant *ν*_0_ for β-PdBi_2_, in analogy to 3D strong topological insulators^30^. For 3D band insulators with inversion symmetry, *ν*_0_ obtained from the parity eigenvalues of filled valence bands at eight time-reversal invariant momenta (TRIM) classifies whether it is a strong topological insulator (*ν*_0_=1) or not (*ν*_0_=0). The bulk β-PdBi_2_ is apparently a metal; nevertheless, here we define a gap in which there is no crossing of the bulk band dispersions through the entire Brillouin zone. By considering this gap, we discuss its topological aspect by calculating *ν*_0_. The calculated bulk bands without and with SOI are shown in Fig. 4a,b, respectively. The valence bands are identified by numbers (from 1st to 10th) as indicated on the right side of respective graphs. The bands are numbered by the energy (*E*) at the Z point. Note that all bands are doubly spin-degenerate. By comparing Fig. 4a,b, we notice that many anticrossings are introduced by SOI, including the ∼0.55 eV gap opening in the green rectangle region where the surface Dirac cone appears. Here we focus on the gap between the 7th and 6th bulk bands, namely gap 7−6, shaded by pink in Fig. 4b. The distribution of the direct gap between the 7th and 6th bands can be evaluated by the joint density of states as a function of the gap energy *E*_g_, defined as 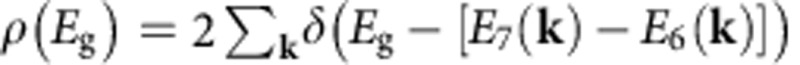. Here, *E*_6_(**k**) and *E*_7_(**k**) represent the respective eigenenergies of the 6th and 7th bands at momentum **k** with **k**=(*k*_*x*_, *k*_*y*_, *k*_*z*_). The result for gap 7−6 is shown in Fig. 4e, which guarantees the minimum value of 0.105 eV gap opening between the 7th and 6th bands through the entire Brillouin zone.

By considering the obtained gap, we discuss its topological aspect by calculating *ν*_0_ in analogy to 3D strong topological insulators. As shown in Fig. 4g, the eight TRIM in the Brillouin zone of β-PdBi_2_ with I4/mmm symmetry are Γ, Z, two X and four N points. Considering these TRIM, *Z*_2_ invariant for the gap between the (*N*+1)-th and *N*-th bulk bands, *ν*_0_(*N*), can be calculated by 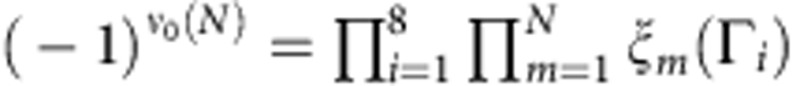, where 
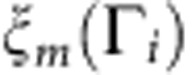
 represents the parity eigenvalue (±1) of the *m*-th band at *i*-th TRIM. Note that since there are even numbers of X and N points, only Γ_*i*_=Γ and Z contribute to the calculation of *ν*_0_(*N*), that is, 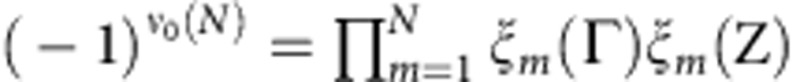. Thus, *ν*_0_ can be calculated by considering solely Γ and Z points, whose symmetries of wavefunctions are listed in Fig. 4d for respective bands. Those indicated by red (black) is of odd (even) parity. We find that gap 7−6 is characterized by *ν*_0_(6)=1, indicating its analogy to 3D strong topological insulators. This requires an odd number of surface states connecting the 7th and 6th bands, to topologically link the bulk β-PdBi_2_ and a vacuum. The observation of spin-helical surface Dirac cone in gap 7−6 clearly represents the characters of such topologically protected surface states.

### Topological surface state crossing *E*_F_

**Figure 5:**
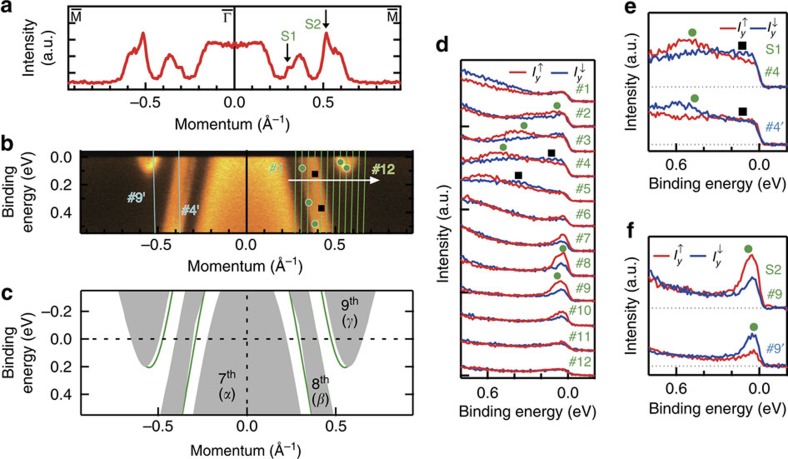
Spin-polarized surface states crossing the Fermi level. (**a**–**c**) Momentum distribution curve obtained by integrating the intensity in the energy window of ±10 meV at the Fermi level, the intensity image and the calculation, respectively, shown along 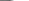
–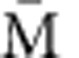. The black arrows in **a** indicate the intensities from two different surface bands denoted by S1 and S2. Green circles (black squares) depicted in **b** are the peak positions of energy- and momentum-distribution curves for surface (bulk) bands. In **c**, surface band dispersions (green) are overlaid to two-dimensional projected bulk bands (grey), namely the 7th (*α*), 8th (*β*) and 9th (*γ*) bands. (**d**) Spin-resolved spectra recorded at momenta #1–12, as shown in **b**, respectively. (**e**,**f**) Spin-resolved spectra for S1 at momenta #4 and #4′ in **b**, and for S2 at momenta #9 and #9′ in **b**, respectively. Red (blue) curves in **d**–**f** show the spin-up (spin-down) component of the intensity for spin-*y*, 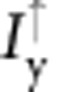 (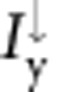). Green circles (black squares) depicted in **d**–**f** are identical to those in **b**.

By further looking at the list of *ν*_0_ in Fig. 4d, we notice *ν*_0_(8)=1 for gap 9−8 shaded by blue in Fig. 4b, which has a minimum gap of 0.127 eV as confirmed by the calculation (Fig. 4f). It suggests that the topological surface states connecting the 9th and 8th bands must exist, where we may observe the effect of superconductivity if located close enough to *E*_F_. To clarify this possibility, the close-up of ARPES image near *E*_F_ is shown with the calculation in Fig. 5b,c. The green curves in Fig. 5c indicate the calculated surface states crossing *E*_F_ separately from the 2D projected bulk bands shaded by grey. They appear at the smaller-*k*_*x*_ side of *β* (8th) and *γ* (9th) bands. Experimentally, the sharp peaks indicative of 2D surface states are observed in momentum distribution curve at *E*_F_, as denoted by S1 and S2 in Fig. 5a. As can be seen in the list of *ν*_0_ in Fig. 4d, S2 should be the topological surface state connecting the 9th and 8th bands, whereas S1 appearing in gap 8−7 must be trivial.

The spin polarization of the topological surface state S2 as well as the trivial surface state S1 is also confirmed experimentally. As shown in Fig. 5d, the *y*-oriented spin polarizations of S1 (#2–5) and S2 (#7–10) along *k*_*x*_ (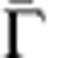
–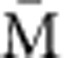) are clearly observed in the spin-resolved spectra. Here, the peak positions for S1 and S2 (bulk *β*) bands are depicted by green circles (black squares). We can see that S1 and S2 are both spin-polarized with spin-up for *k*_*x*_>0, whereas they get inverted for *k*_*x*_<0 (Fig. 5e,f) as required by the time-reversal symmetry. These clearly indicate that both topological and trivial surface states crossing *E*_F_ possess the in-plane spin polarizations.

## Discussion

**Figure 6:**
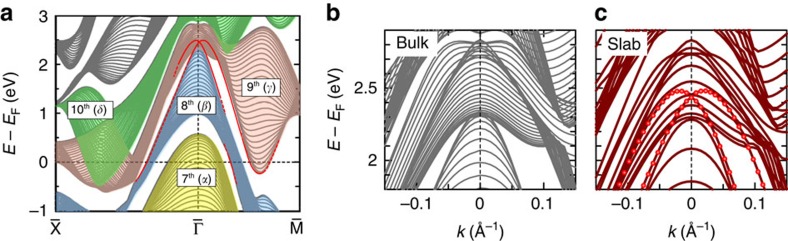
Band crossing of the topological surface state. (**a**) Two-dimensional projected bulk bands and the surface state bands obtained by the calculation. The energy (*E*) relative to the Fermi level (*E*_F_) is plotted along 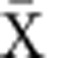
–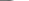 and 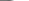
–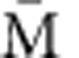. Those crossing the Fermi level are painted by colours; yellow for 7th (*α*), blue for 8th (*β*), pink for 9th (*γ*) and green for the 10th (*δ*) bands. The surface state bands crossing the Fermi level are depicted by the red curves. (**b**,**c**) The calculated band dispersions for the bulk PdBi_2_ and for the slab of 11 PdBi_2_ layers, respectively, magnified near the band crossing point. *E*–*E*_F_ for respective bands are plotted as a function of momentum *k*. The bands at the topmost surface are highlighted by red markers in **c**.

The *Z*_2_ analysis shows that odd number of gapless surface states in gap 9−8, connecting the 9th and 8th bands, must exist between 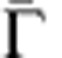 and 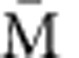. To confirm whether the experimentally observed S2 indeed corresponds to this topological surface state, we need to carefully look at the slab calculation since S2 crosses *E*_F_ and extends to the unoccupied state. By tracking the calculated data from 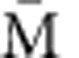 towards 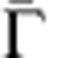 (Fig. 6a), we first notice that S2 is derived from the local minimum of the 9th (*γ*) band. S2 then crosses *E*_F_ and reaches up to *E*–*E*_F_=2 eV without merging into the bulk states. At 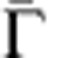, although it gets overlapped with 2D projected bulk bands, we can distinguish S2 forming a Rashba-like crossing point at *E*–*E*_F_=2.4 eV. After the crossing, S2 band eventually gets merged into the 8th (*β*) band. It thus shows that S2 indeed connects the 9th and 8th bands. The crossing of S2 surface band at 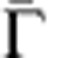 is more clearly seen, by comparing the 2D projected bulk (Fig. 6b) and the slab (Fig. 6c) calculations magnified near the crossing point. The crossing of the S2 surface band at 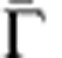 is distinguished in Fig. 6c, by following the eigenenergies highlighted with the red markers. Note that no such crossing exists for the calculation of bulk in Fig. 6b. S2 thus possesses a similarity to the Dirac cone that connects the gap with the crossing at 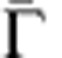, and is indeed a topologically protected surface state.

Here we note that the spin-polarized topological S2 and the surface Dirac cone are both derived as a consequence of SOI, but in different processes. For the case of S2 in gap 9−8, we see that *ν*_0_ changes to 1 by including the SOI. It thus indicates the band inversion associated with the 8th, 9th and 10th bands occurring at Γ (see Fig. 4c,d) induced by the SOI. This situation is fairly similar to the topological phase transition being discussed in 3D strong topological insulators^31^. For the surface Dirac cone in gap 7−6, on the other hand, *ν*_0_=1 is realized already in the non-relativistic case (Fig. 4c), due to the inversion of A_1g_ and A_2u_ bands introduced by Bi6*p*–Pd4*d* mixing. This non-relativistic situation should be rather similar to the 3D Dirac semimetals^32^,^33^, as represented by the bulk Dirac points appearing along Z–M and Z–X (Fig. 4a), which may accompany the spin-degenerate surface states (Fermi arcs). The role of the SOI in this case is the gap opening at these bulk Dirac points, giving rise to the spin-polarized surface Dirac cone connecting the gap edges.

The next future step for β-PdBi_2_ should be the direct elucidation of the superconducting state. Low-temperature ultrahigh-resolution ARPES will surely be a strong candidate for such investigation^34^,^35^. There may be a chance to observe non-trivial superconducting excitations, by selectively focusing on the surface and bulk band dispersions as experimentally presented in Bi_2_Se_3_/NbSe_2_ thin film^34^. Scanning tunnelling microscope/spectroscopy, on the other hand, can locally probe the superconducting state around the vortex cores. As theoretically suggested, it may capture the direct evidence of Majorana mode^4^,^11^,^36^,^37^. We should note that β-PdBi_2_ will also provide a solid platform for bulk measurements such as thermal conductivity and nuclear magnetic resonance, which are expected to give some information on the odd-parity superconductivity^18^,^19^. It may thus contribute to making the realm of superconducting topological materials, and pave the way to various new findings such as the direct observation of Majorana fermions dispersion and/or surface Andreev bound states^36^,^37^, clarification of its relation to the possible odd-parity superconductivity^11^,^17^ and bulk-surface mixing effect^36^,^38^.

## Methods

### Crystal growth

Single crystals of β-PdBi_2_ were grown by a melt growth method. Pd and Bi at a molar ratio of 1:2 were sealed in an evacuated quartz tube, pre-reacted at high temperature until it completely melted and mixed. Then, it was again heated up to 900 °C, kept for 20 h, cooled down at a rate of 3 °C h^−1^ down to 500 °C and rapidly quenched into cold water. The obtained single crystals had good cleavage, producing flat surfaces as large as ∼1 × 1 cm^2^. The resistivity shown in Fig. 1b and the magnetic susceptibility shown in Fig. 1c exhibit the clear superconducting transition at *T*_c_=5.3 K.

### Angular-resolved photoemission spectroscopy (ARPES)

ARPES measurement with the HeIα light source (21.2 eV) were made at the Department of Applied Physics, The University of Tokyo, using a VUV5000 He-discharge lamp and an R4000 hemispherical electron analyzer (VG-Scienta). The total energy resolution was set to 10 meV. Samples were cleaved *in situ* at around room temperature and measured at 20 K.

### Spin- and angular-resolved photoemission spectroscopy (SARPES)

SARPES with the HeIα light source (21.2 eV) was performed at the Efficient SPin REsolved SpectroScOpy (ESPRESSO) end station attached to the APPLE-II-type variable polarization undulator beamline (BL-9B) at the Hiroshima Synchrotron Radiation Center (HSRC)^29^. The analyzer of this system consists of two sets of very-low-energy electron diffraction spin detectors, thus enabling the detection of the electron spin orientation in three dimension^39^. The angular resolution was set to ±1.5° and the total energy resolution was set to 35 meV. Samples were cleaved *in situ* at around room temperature and measured at 20 K.

### Band calculations

First-principles electronic structure calculations within the framework of the density functional theory were performed using the full-potential linearized augmented plane-wave method as implemented in the WIEN2k code^40^, with the generalized gradient approximation of Perdew, Burke and Ernzerhof exchange-correlation function^41^. SOI was included as a second variational step with a basis of scalar-relativistic eigenfunctions.

The experimental crystal data (*a*=3.362 Å, *c*=12.983 Å, *z*(Bi)=0.363) were used for the bulk calculations. The (001) surface was simulated by a slab model; a stacking of 11 PdBi_2_-triple layers along the *c* axis with a 15 Å of vacuum layer, forming a tetragonal crystal structure of space group *P*4/mmm with the lattice constants of *a*=3.362 Å and *c*=83.423 Å.

The plane-wave cutoff energy was set to *R*_MT_*K*_max_=9, where the muffin tin radii are *R*_MT_=2.5 a.u. for both Bi and Pd. The Brillouin zone was sampled with the Monkhorst-Pack scheme^42^ with momentum grids finer than *Δk*=0.02 Å^−1^ (for example, a Γ-centred 38 × 38 × 38 *k*-point mesh was used for the Fermi surface visualization, corresponding to *Δk*=0.009 Å^−1^).

## Additional information

**How to cite this article:** Sakano, M. *et al*. Topologically protected surface states in a centrosymmetric superconductor β-PdBi_2_. *Nat. Commun.* 6:8595 doi: 10.1038/ncomms9595 (2015).

## Supplementary Material

Supplementary Information Supplementary Figure 1, Supplementary Note 1 and Supplementary References

## References

[b1] Hasan M. Z. & Kane C. L. Colloquium: topological insulators. Rev. Mod. Phys. 82, 3045–3067 (2010).

[b2] Qi X.-L. & Zhang S.-C. Topological insulators and superconductors. Rev. Mod. Phys. 83, 1057–1110 (2011).

[b3] Schnyder A. P., Ryu S., Furusaki A. & Ludwig A. W. W. Classification of topological insulators and superconductors in three spatial dimensions. Phys. Rev. B 78, 195125 (2008).

[b4] Fu L. & Kane C. L. Superconducting proximity effect and majorana fermions at the surface of a topological insulator. Phys. Rev. Lett. 100, 096407 (2008). 18352737 10.1103/PhysRevLett.100.096407

[b5] Wilczek F. Majorana returns. Nat. Phys. 5, 614–618 (2009).

[b6] Tanaka Y., Sato M. & Nagaosa N. Symmetry and topology in superconductors: odd-frequency pairing and edge states. J. Phys. Soc. Jpn 81, 011013 (2012).

[b7] Alicea J. New directions in the pursuit of Majorana fermions in solid state systems. Rep. Prog. Phys. 75, 075602 (2012). 10.1088/0034-4885/75/7/076501 22790778

[b8] Beenakker C. W. J. Search for Majorana fermions in superconductors. Annu. Rev. Condens. Matter Phys. 4, 113–116 (2013).

[b9] Hor Y. S. . Superconductivity in Cu_*x*_Bi_2_Se_3_ and its Implications for Pairing in the Undoped Topological Insulator. Phys. Rev. Lett. 104, 057001 (2010). 20366785 10.1103/PhysRevLett.104.057001

[b10] Kriener M., Segawa K., Ren Z., Sasaki S. & Ando Y. Bulk superconducting phase with a full energy gap in the doped topological insulator Cu_*x*_Bi_2_Se_3_. Phys. Rev. Lett. 106, 127004 (2011). 21517345 10.1103/PhysRevLett.106.127004

[b11] Fu L. & Berg E. Odd-parity topological superconductors: theory and application to Cu_*x*_Bi_2_Se_3_. Phys. Rev. Lett. 105, 097001 (2010). 20868184 10.1103/PhysRevLett.105.097001

[b12] Sasaki S. . Topological superconductivity in Cu_*x*_Bi_2_Se_3_. Phys. Rev. Lett. 107, 217001 (2011). 22181913 10.1103/PhysRevLett.107.217001

[b13] Sasaki S. . Odd-parity pairing and topological superconductivity in a strongly spin-orbit coupled semiconductor. Phys. Rev. Lett. 109, 217004 (2012). 23215610 10.1103/PhysRevLett.109.217004

[b14] Zhang J. L. . Pressure-induced superconductivity in topological parent compound Bi_2_Te_3_. Proc. Natl Acad. Sci. USA 108, 24–28 (2011). 21173267 10.1073/pnas.1014085108 PMC3017179

[b15] Zhu J. . Superconductivity in topological insulator Sb_2_Te_3_ induced by pressure. Sci. Rep. 3, 2016 (2013). 23783511 10.1038/srep02016 PMC3687246

[b16] Levy N. . Experimental evidence for s-wave pairing symmetry in superconducting Cu_*x*_Bi_2_Se_3_ single crystals using a scanning tunneling microscope. Phys. Rev. Lett. 110, 117001 (2013). 25166563 10.1103/PhysRevLett.110.117001

[b17] Mizushima T., Yamakage A., Sato M. & Tanaka Y. Dirac-fermion-induced parity mixing in superconducting topological insulators. Phys. Rev. B 90, 184516 (2014).

[b18] Zocher B. & Rosenow B. Surface states and local spin susceptibility in doped three-dimensional topological insulators with odd-parity superconducting pairing symmetry. Phys. Rev. B 87, 155138 (2013).

[b19] Nagai Y., Nakamura H. & Machida M. Rotational isotropy breaking as proof for spin-polarized Cooper pairs in the topological superconductor Cu_x_Bi_2_Se_3_. Phys. Rev. B 86, 094507 (2012).

[b20] Lin H. . Half-Heusler ternary compounds as new multifunctional experimental platforms for topological quantum phenomena. Nat. Mater. 9, 546–549 (2010). 20512153 10.1038/nmat2771

[b21] Liu C. . Metallic surface electronic state in half-Heusler compounds *R*PtBi (*R*=Lu, Dy, Gd). Phys. Rev. B 83, 205133 (2011).

[b22] Xu R., de Groot R. A. & van der Lugt W. The electrical resistivities of liquid Pd-Bi alloys and the band structure of crystalline beta-PdBi_2_ and PdBi. J. Phys. Condens. Matter 4, 2389–2395 (1992).

[b23] Imai Y. . Superconductivity at 5.4 K in β-Bi_2_Pd. J. Phys. Soc. Jpn 81, 113708 (2012).

[b24] Shein I. R. & Ivanovskii A. L. Electronic band structure and Fermi surface of tetragonal low-temperature superconductor Bi_2_Pd as predicted from first principles. J. Supercond. Nov. Magn. 26, 1–4 (2013).

[b25] Mondal M. . Andreev bound state and multiple energy gaps in the noncentrosymmetric superconductor BiPd. Phys. Rev. B 86, 094520 (2012).

[b26] Sun Z. . Dirac surface states and nature of superconductivity in noncentrosymmetric BiPd. Nat. Commun. 6, 6633 (2015). 25818338 10.1038/ncomms7633 PMC4389226

[b27] Chen Y. L. . Experimental realization of a three-dimensional topological insulator, Bi_2_Te_3_. Science 325, 178–181 (2009). 19520912 10.1126/science.1173034

[b28] Fu L. Hexagonal warping effects in the surface states of the topological insulator Bi_2_Te_3_. Phys. Rev. Lett. 103, 266801 (2009). 20366328 10.1103/PhysRevLett.103.266801

[b29] Okuda T. . Efficient spin resolved spectroscopy observation machine at Hiroshima Synchrotron Radiation Center. Rev. Sci. Instrum. 82, 103302 (2011). 22047286 10.1063/1.3648102

[b30] Fu L. & Kane C. L. Topological insulators with inversion symmetry. Phys. Rev. B 76, 045302 (2007).

[b31] Xu S.-Y. . Topological phase transition and texture inversion in a tunable topological insulator. Science 332, 560–564 (2011). 21454752 10.1126/science.1201607

[b32] Neupane M. . Observation of a three-dimensional topological Dirac semimetal phase in high-mobility Cd_3_As_2_. Nat. Commun. 5, 3786 (2014). 24807399 10.1038/ncomms4786

[b33] Liu Z. K. . A stable three-dimensional topological Dirac semimetal Cd_3_As_2_. Nat. Mater. 13, 677–681 (2014). 24859642 10.1038/nmat3990

[b34] Okazaki K. . Octet-line node structure of superconducting order parameter in KFe_2_As_2_. Science 337, 1314–1317 (2012). 22984065 10.1126/science.1222793

[b35] Xu S.-Y. . Momentum-space imaging of Cooper pairing in a half-Dirac-gas topological superconductor. Nat. Phys. 10, 943–950 (2014).

[b36] Hosur P., Ghaemi P., Mong R. S. K. & Vishwanath A. Majorana modes at the ends of superconductor vortices in doped topological insulators. Phys. Rev. Lett. 107, 097001 (2011). 21929261 10.1103/PhysRevLett.107.097001

[b37] Hsieh T. H. & Fu L. Majorana fermions and exotic surface Andreev bound states in topological superconductors: application to Cu_*x*_Bi_2_Se_3_. Phys. Rev. Lett. 108, 107005 (2012). 22463445 10.1103/PhysRevLett.108.107005

[b38] Bergman D. L. & Refael G. Bulk metals with helical surface states. Phys. Rev. B 82, 195417 (2010).

[b39] Okuda T., Miyamoto K., Kimura A., Namatame H. & Taniguchi M. A double VLEED spin detector for high-resolution three dimensional spin vectorial analysis of anisotropic Rashba spin splitting. J. Electron Spectrosc. Relat. Phenom. 201, 23–20 (2015).

[b40] Blaha P., Schwarz K., Sorantin P. & Trickey S. B. Full-potential, linearized augmented plane wave programs for crystalline systems. Comp. Phys. Commun. 59, 399–415 (1990).

[b41] Perdew J. P., Burke K. & Ernzerhof M. Generalized gradient approximation made simple. Phys. Rev. Lett. 77, 3865–3868 (1996). 10062328 10.1103/PhysRevLett.77.3865

[b42] Monkhorst H. J. & Pack J. D. Special points for Brillouin-zone integrations. Phys. Rev. B 13, 5188–5192 (1976).

