# Supplementary material for: Topologically protected surface states in a centrosymmetric superconductor β-PdBi2
Source: Nat Commun. 2015 Oct 13;6:8595. doi: 10.1038/ncomms9595 (PMC4633943; doi:10.1038/ncomms9595)
Supplement: Supplementary Information — Supplementary Figure 1, Supplementary Note 1 and Supplementary References [file ncomms9595-s1.pdf]

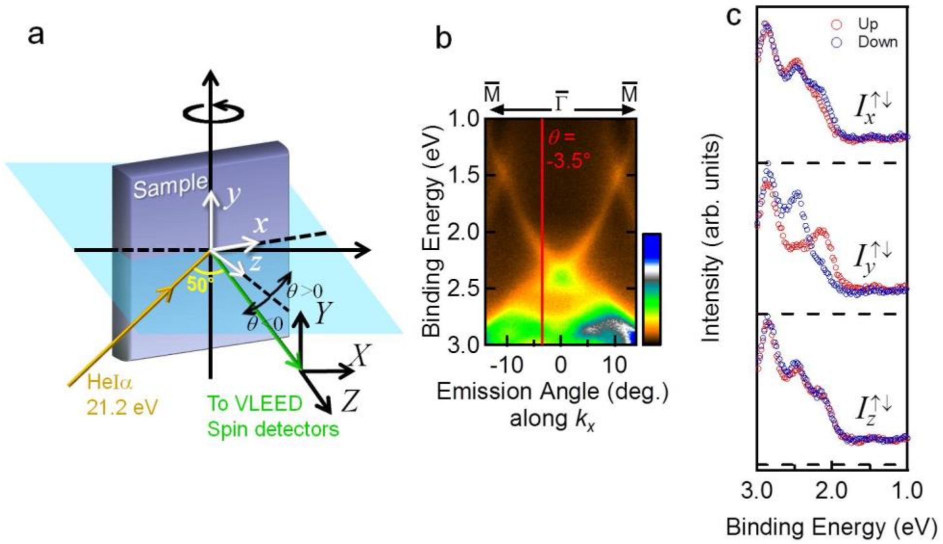

Supplementary Figure 1: Spin polarization of surface Dirac-cone band

a, Schematic of spin- and angular-resolved photoemission spectroscopy (SARPES) experimental geometry using the HeIα light source (21.2 eV) and very low energy electron diffraction (VLEED) spin detectors. **b,** Angular-resolved photoemission (ARPES) image plotted as functions of the emission angle θ and the binding energy, showing the surface Dirac-cone. **c,** Spin-resolved energy distribution curves recorded at $\theta = -3.5°$ for x, y, z spin orientations, respectively. Red (blue) markers indicate the intensities for spin-up (spin-down) components, $I_x^{\uparrow}$ ($I_x^{\downarrow}$), $I_y^{\uparrow}$ ($I_y^{\downarrow}$), and $I_z^{\uparrow}$ ($I_z^{\downarrow}$).

Supplementary Note 1. Spin- and angular- resolved photoemission spectroscopy (SARPES)

SARPES with the HeIα light source (21.2 eV) was performed at the Efficient SPin REsolved SpectroScOpy (ESPRESSO) end station attached to the APPLE-II type variable polarization undulator beam line (BL-9B) at the Hiroshima Synchrotron Radiation Center (HSRC)[1]. The analyzer of this system consists of 2 sets of VLEED (Very low energy electron diffraction) spin detectors, thus enabling the detection of the electron spin orientation in 3 dimension[2]. The angular resolution was set to ±1.5° and the total energy resolution was set to 35 meV. Samples were cleaved *in situ* at around room temperature and measured at 20 K.

The geometry of the measurement is shown in Supplementary Figure 1a. Here, *x*, *y*, *z* represent the axes fixed at the β-$PdBi_2$ sample, where *x* and *y* are taken along the in-plane nearest Pd-Pd directions and *z* along the stacking direction. *X*, *Y*, *Z* are the coordinate system fixed at the spin detector system. The magnetization of the targets in the two VLEED spin detectors can be selectively aligned along *X*, *Z*, and *Y*, *Z*, respectively, by using electric coils. The acquisition of the spin polarization along the β-$PdBi_2$ crystal axes, *x*, *y*, *z*, is available by the following procedures.

The spin polarization of the electron at the spin detector, e.g. P_Y for *Y*-direction, is obtained by $P_Y = \frac{1}{S_{eff}} \frac{I_Y^+ - I_Y^-}{I_Y^+ + I_Y^-}$, where $S_{eff} = 0.32$ is the effective Sherman function and I_Y^+, I_Y^- are the raw ARPES spectra recorded with the target magnetization of plus and minus along *Y*-direction. P_X and P_Z are similarly obtained from $I_X^{+,-}$ and $I_Z^{+,-}$, respectively. The spin-up and spin-down spectra, $I_X^{\uparrow,\downarrow}$, $I_Y^{\uparrow,\downarrow}$, $I_Z^{\uparrow,\downarrow}$, are calculated by $I_Y^{\uparrow,\downarrow} = (1 \pm P_Y)\frac{I_Y^+ + I_Y^-}{2}$, and etc.

The spin polarization P_x, P_y, P_z and the SARPES spectra $I_x^{\uparrow,\downarrow}$, $I_y^{\uparrow,\downarrow}$, $I_z^{\uparrow,\downarrow}$ projected along the *x*, *y*, *z* crystal axes are related to P_X, P_Y, P_Z and $I_X^{\uparrow,\downarrow}$, $I_Y^{\uparrow,\downarrow}$, $I_Z^{\uparrow,\downarrow}$ by

$$\begin{pmatrix} P_x \\ P_y \\ P_z \end{pmatrix} = \begin{pmatrix} \cos\theta & 0 & -\sin\theta \\ 0 & 1 & 0 \\ \sin\theta & 0 & \cos\theta \end{pmatrix} \begin{pmatrix} P_X \\ P_Y \\ P_Z \end{pmatrix}, \quad \begin{pmatrix} I_x^{\uparrow,\downarrow} \\ I_y^{\uparrow,\downarrow} \\ I_z^{\uparrow,\downarrow} \end{pmatrix} = \begin{pmatrix} \cos\theta & 0 & -\sin\theta \\ 0 & 1 & 0 \\ \sin\theta & 0 & \cos\theta \end{pmatrix} \begin{pmatrix} I_X^{\uparrow,\downarrow} \\ I_Y^{\uparrow,\downarrow} \\ I_Z^{\uparrow,\downarrow} \end{pmatrix},$$

where θ is the emission angle of the electron. $I_x^{\uparrow,\downarrow}$, $I_y^{\uparrow,\downarrow}$, and $I_z^{\uparrow,\downarrow}$ thus obtained are shown in Supplementary Figure 1c for $\theta = -3.5°$ as indicated in Supplementary Figure 1b. Note that for *y*-component which is discussed in the main text, the simple relation $I_y^{\uparrow,\downarrow} = I_Y^{\uparrow,\downarrow}$ holds regardless of θ.

Supplementary References

1. Okuda, T., Miyamoto, K., Miyahara, H., Kuroda, K., Kimura, A. & Namatame, H. Efficient spin resolved spectroscopy observation machine at Hiroshima Synchrotron Radiation Center. *Rev. Sci. Instrum.* **82**, 103302 (2011).
2. Okuda, T., Miyamoto, K., Kimura, A., Namatame, H. & Taniguchi, M. A double VLEED spin detector for high-resolution three dimensional spin vectorial analysis of anisotropic Rashba spin splitting. *J. Electron Spectrosc. Relat. Phenom.* **201**, 23-20 (2015).
